# Low-dose Actinomycin-D treatment re-establishes the tumoursuppressive function of P53 in *RELA*-positive ependymoma

**DOI:** 10.18632/oncotarget.11452

**Published:** 2016-08-20

**Authors:** Theophilos Tzaridis, Till Milde, Kristian W. Pajtler, Sebastian Bender, David T. W. Jones, Simone Müller, Andrea Wittmann, Magdalena Schlotter, Andreas E. Kulozik, Peter Lichter, V. Peter Collins, Olaf Witt, Marcel Kool, Andrey Korshunov, Stefan M. Pfister, Hendrik Witt

**Affiliations:** ^1^ Division of Pediatric Neurooncology (B062), German Cancer Consortium (DKTK), German Cancer Research Center (DKFZ), Heidelberg, Germany; ^2^ Department of Pediatric Oncology, Hematology and Immunology, Children's Hospital, University of Heidelberg, Heidelberg, Germany; ^3^ Clinical Cooperation Unit Pediatric Oncology (G340), German Cancer Research Center (DKFZ), Heidelberg, Germany; ^4^ National Center for Tumor diseases (NCT), Clinical Trial Center, Heidelberg, Germany; ^5^ Department of Cardiology, Angiology and Pulmology, University of Heidelberg, Heidelberg, Germany; ^6^ Division of Molecular Genetics (B060), German Cancer Research Center (DKFZ) and German Cancer Consortium (DKTK), Heidelberg, Germany; ^7^ Division of Molecular Histopathology, Department of Pathology, University of Cambridge, Cambridge UK; ^8^ Clinical Cooperation Unit Neuropathology (G380), German Cancer Research Center (DKFZ) and German Cancer Consortium (DKTK), Heidelberg, Germany; ^9^ Department of Neuropathology, Heidelberg University Hospital, Heidelberg, Germany

**Keywords:** ependymoma, p53, RELA, Actinomycin-D

## Abstract

Ependymomas in children can arise throughout all compartments of the central nervous system (CNS). Highly malignant paediatric ependymoma subtypes are Group A tumours of the posterior fossa (PF-EPN-A) and *RELA*-fusion positive (ST-EPN-RELA) tumours in the supratentorial compartment. It was repeatedly reported in smaller series that accumulation of p53 is frequently observed in ependymomas and that immunohistochemical staining correlates with poor clinical outcome, while *TP53* mutations are rare. Our *TP53* mutation analysis of 130 primary ependymomas identified a mutation rate of only 3%. Immunohistochemical analysis of 398 ependymomas confirmed previous results correlating the accumulation of p53 with inferior outcome. Among the p53-positive ependymomas, the vast majority exhibited a RELA fusion leading to the hypothesis that p53 inactivation might be linked to RELA positivity.

In order to assess the potential of p53 reactivation through MDM2 inhibition in ependymoma, we evaluated the effects of Actinomycin-D and Nutlin-3 treatment in two preclinical ependymoma models representing the high-risk subtypes PF-EPN-A and ST-EPN-RELA. The IC-50 of the agent as determined by metabolic activity assays was in the lower nano-molar range (0.2–0.7 nM). Transcriptome analyses of high-dose (100 nM), low-dose (5 nM) and non-treated cells revealed re-expression of p53 dependent genes including *p53 upregulated modulator of apoptosis* (*PUMA*) after low-dose treatment. At the protein level, we validated the Actinomycin-D induced upregulation of PUMA, and of p53 interaction partners MDM2 and p21. Proapoptotic effects of low-dose application of the agent were confirmed by flow cytometry. Thus, Actinomycin-D could constitute a promising therapeutic option for ST-EPN-RELA ependymoma patients, whose tumours frequently exhibit p53 inactivation.

## INTRODUCTION

Ependymomas are glial brain tumours probably arising from the subventricular zone and accounting for approximately 12% of all paediatric intracranial malignancies [1, 2]. Based on previously reported results of chemotherapeutic regimens across several studies, a clear therapeutic efficacy of this treatment modality has yet to be demonstrated [3], leaving complete resection followed by radiotherapy the only curative and indispensable therapeutic option for these tumours. Complete tumour resection has been suggested as one of the most important favourable prognostic features in several previous studies [4, 5]. The beneficial effect of adjuvant radiotherapy has frequently been reported in clinical studies and is nowadays realisable for patients even down to the age of one year or lower [6, 7], however at the cost of increased morbidity due to radiation-induced sequelae [8]. Lepto-meningeal dissemination occurring with an incidence of 9% to 20% [9], as well as local tumour progression comprise the main clinical challenges leading to a 5 year overall survival rate of only around 60% for all ependymomas [10]. To achieve the goal of increasing the overall survival rates, while simultaneously reducing serious long-term morbidity, the fact that ependymomas in different compartments are actually distinct diseases exhibiting pronounced intertumoural heterogeneity needs to be taken into account [11]. According to the recent WHO grading system, differentiation criteria between grade II and III ependymomas demonstrate great uncertainty, thus their reliability as prognostic factors remains doubtful [6, 12, 13].

Based on transcriptome classification, two distinct subtypes of posterior fossa ependymomas were first described [14]. Group A tumours were shown to be associated with inferior prognosis and exhibited an upregulation of classical tumour-signalling pathways some of which lead to defects in the p53 cascade. Recently, the fusion oncogene *RELA*-*C11orf95,* leading to constitutively active NF-kappaB signalling, was identified as a centrally important molecular driver event in supratentorial ependymomas [15]. Notably, aberrant NF-kappaB activity has been shown to induce MDM2 expression, thereby resulting in p53 inactivation [16]. Finally, a comprehensive worldwide molecular classification study across the whole spectrum of ependymal brain tumours of all anatomic locations and age groups resulted in a proposed molecular classification system distinguishing nine distinct molecular subgroups based on DNA methylation fingerprints [11]. Within paediatric ependymoma cohorts, four molecular subtypes represent the vast majority of cases including the infratentorial Group A PF-EPN-A and Group B (PF-EPN-B), as well as supratentorial *RELA*-fusion positive (ST-EPN-RELA) and *YAP1*-fusion positive (ST-EPN-YAP1) ependymomas with Group A and ST-EPN-RELA being the most aggressive subtypes.

Before the era of molecular subgrouping, immunopositivity for p53 was established as a marker for poor outcome [17, 18]. Interestingly, despite p53 accumulation, *TP53* mutations were reported to be extremely rare in ependymomas [19, 20]. Thus, along with the observation of a low apoptosis rate in tumour cells, several studies concluded that p53 is functionally impaired in ependymomas [21, 22]. Furthermore, it was shown that p14/ARF downregulation, frequently caused by *CDKN2A* deletion, is associated with biologically aggressive tumours and p53 accumulation [23]. Milde et al. lately generated a supratentorial paediatric ependymoma cell model (EP1NS) harbouring a *RELA*-fusion, as well as a homozygous *CDKN2A* deletion. This latter change was shown to be associated with unfavourable prognosis in several retrospective cohorts [24–26]. An attractive pharmacological strategy in tumours with p53 accumulation without the presence of a *TP53* mutation might be the reactivation of wild-type p53 via inhibition of MDM2 [27]. Important agents of this category include Actinomycin-D, and Nutlin-type inhibitors. Actinomycin-D is a well known chemotherapeutic drug for the treatment of paediatric brain tumours, which was applied within clinical trials of atypical teratoid/rhabdoid tumours [28] and low grade gliomas [29]. Currently, several other clinical trials in paediatric oncology include Actinomycin-D as part of a combination chemotherapy, e.g. for Wilms tumour (ClinicalTrials.gov ID: NCT00047138), Ewing sarcoma (ClinicalTrials.gov ID: NCT00541411) and rhabdomyosarcoma (ClinicalTrials.gov ID: NCT00002995). It has already been shown that low-dose Actinomycin-D restores the function of p53 by mediating apoptosis in various *TP53* wildtype tumour cell lines [30–32]. Nutlin-3 comprises a cis-imidazoline small-molecule compound, which binds and inhibits MDM2 thereby increasing levels of stable p53 *in vitro* and *in vivo* [33].

In this study, we demonstrate MDM2 overactivity through either constitutive NF-κB activation or homozygous *CDKN2A* deletion as a plausible mechanism of p53 abrogation and report Actinomycin-D induced p53 reactivation at RNA, protein and functional levels in preclinical high-risk ependymoma models. Furthermore, we show that these effects are mediated by low-dose and to a less extent by high-dose concentrations of the agent. Application of Nutlin-3 showed only partial efficacy in treated ependymoma cells. Finally, we prove the specific efficacy of Actinomycin-D for this tumour by comparing the treatment of ependymoma cells to medulloblastoma and human fetal neural stem cells.

## RESULTS

### Alterations of p53 in primary ependymomas and cell lines EP1NS and SJ-BT57

**Figure 1:**
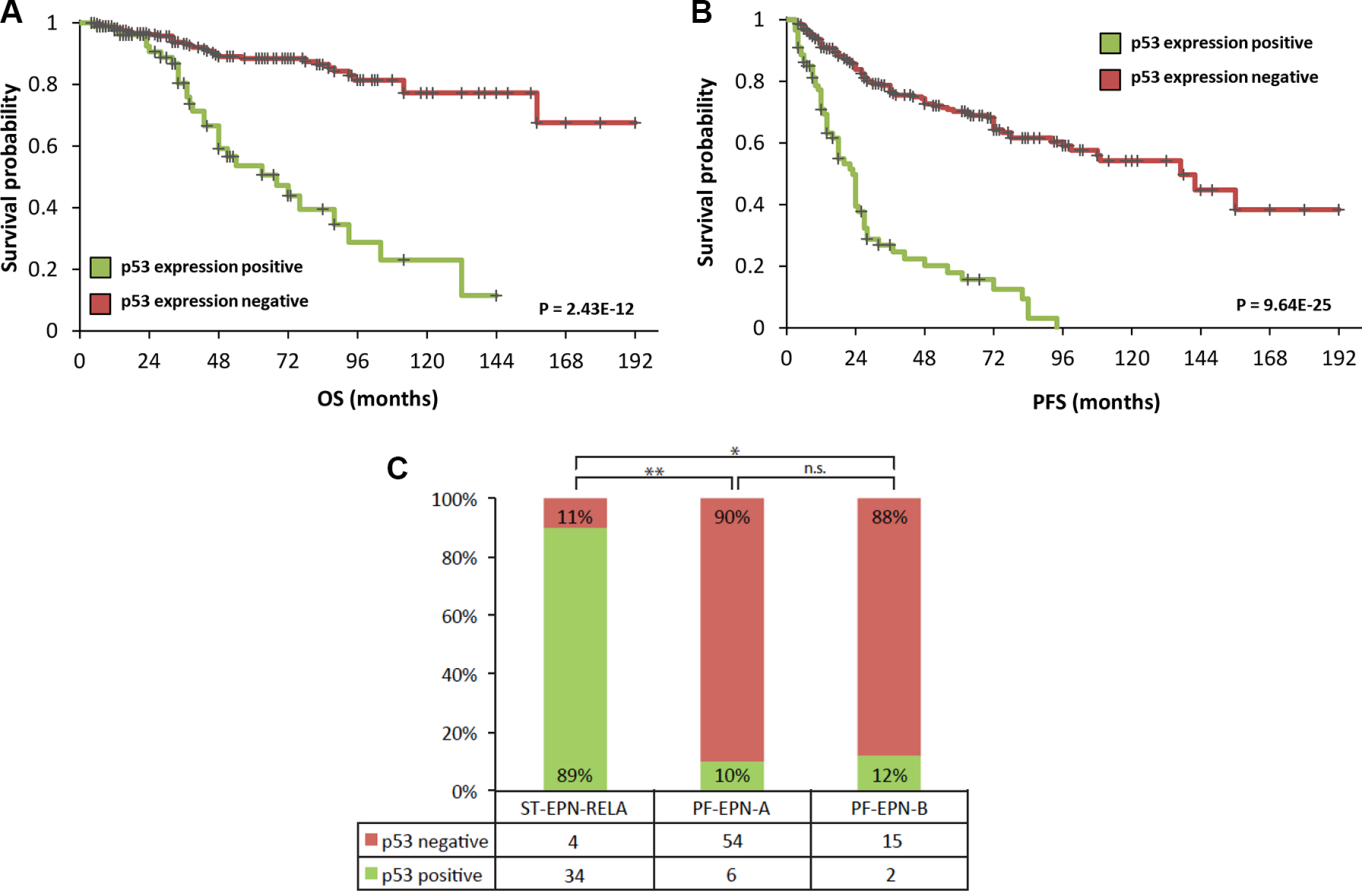
TMA data of 398 primary ependymomas showing a correlation of high p53 expression with worse progression-free (A) and overall (B) survival Statistical significance was determined by a log-rank test. Furthermore, correlation between p53 expression and *RELA*-fusion positive supratentorial ependymomas with 89% (34/38) of supratentorial *RELA*-fusion positive tumours exhibiting p53 positivity (**C**). On the contrary, only a small subset of infratentorial Group A (6/60) ependymomas and two of Group B tumours (2/17) show p53 accumulation (*P = 3.63E-8, **P = 7.61E-16; by *Fisher's exact test*).

Consistent with previous results, a *TP53* mutation rate of only 3% (4/130) was detected in primary ependymomas (Supplementary Table S1). Genome wide mutation analyses of the two ependymoma cell lines (EP1NS and SJ-BT57) also showed the absence of *TP53* mutations (data not shown). Previous molecular characterisation of the cell lines EP1NS and SJ-BT57 using the Illumina 450k DNA methylation array revealed subgroup affiliation with ST-EPN-RELA and PF-EPN-A respectively (data not shown). While RNA-sequencing detected the prototypic *RELA-C11orf95* fusion in EP1NS cells, it was absent in SJ-BT57 cells (data not shown). Additional well-established and representative ependymoma cell lines are still lacking to date. At the protein level, a high overall incidence of p53 accumulation in primary tumours was detected by immunohistochemistry (22%; *n* = 88/398, Supplementary Figure S1A). In this cohort, p53 accumulation was associated with inferior progression-free and overall survival, which is in line with previous reports (Figure 1A and 1B) [17, 18]. When correlating p53 status with subgroup information, which was available for 102 samples (ST-EPN-RELA (*n* = 38), PF-EPN-A (*n* = 60) and PF-EPN-B (*n* = 17)), it was striking that 89% (*n* = 34/38) of the supratentorial *RELA*-positive tumours exhibited p53 accumulation (Figure 1C). Conversely, out of the infratentorial tumours, only 10% (*n* = 6/60) of the Group A ependymomas were p53-positive, and only 12% (*n* = 2/15) of Group B tumours showed p53-immunopositivity. No significant correlation was observed between p53 positivity and relapse or mortality in *RELA*-positive ependymomas (Supplementary Figure S1D). However, we noticed a trend of inferior outcome in p53-positive tumours. This could possibly be attributed to the low number of p53-negative RELA ependymomas (*n* = 4/38).

With respect to the cell lines, p53 accumulation was detected in mouse paraffin sections of orthotopical ependymomas, as well as *in vitro* in both cases, however the protein levels showed different patterns (Supplementary Figure S1B, S1C). While p53-staining was moderate in EP1NS cells with about 20% positive cells, SJ-BT57 cells showed a massive protein accumulation, possibly due to a selection advantage of SJ-BT57 growth *in vitro*. These data along with the above-mentioned reports about decreased apoptosis in p53-positive ependymoma cells lead to the hypothesis that p53 may be inactivated by mechanisms other than mutation in these tumours [21, 22].

Since direct molecular modifications of p53 were exceedingly rare, alternative p53-associated aberrations were considered. A certain proportion of high-risk supratentorial ependymomas, including a fraction of ST-EPN-RELA including the EP1NS cells, are known to harbour homozygous deletions of *CDKN2A* (Milde et al., 2011; Pajtler et al., 2015). In our TMA cohort, 4% (*n* = 6/139) of supratentorial ependymomas contained this genomic aberration and all of these stained positive for p53. It is well established that the p14/ARF protein, encoded by *CDKN2A,* directly inhibits one of the most important antagonists of p53, MDM2. Since the EP1NS cells exhibit a homozygous *CDKN2A* deletion, we sought to confirm the absence of p14. Indeed, p14 was not detected in the EP1NS cells, whereas it was present in several primary PF-EPN-A and PF-EPN-B ependymomas (Supplementary Figure S2). Therefore, we propose the absence of p14-mediated inhibition of MDM2 as one alternative plausible pathway leading to abrogation of the p53 pathway in addition to constitutive NF-κB activation.

### Therapeutic effects of Actinomycin-D and Nutlin-3 on metabolic activity of cells including re-expression of p53

**Figure 2:**
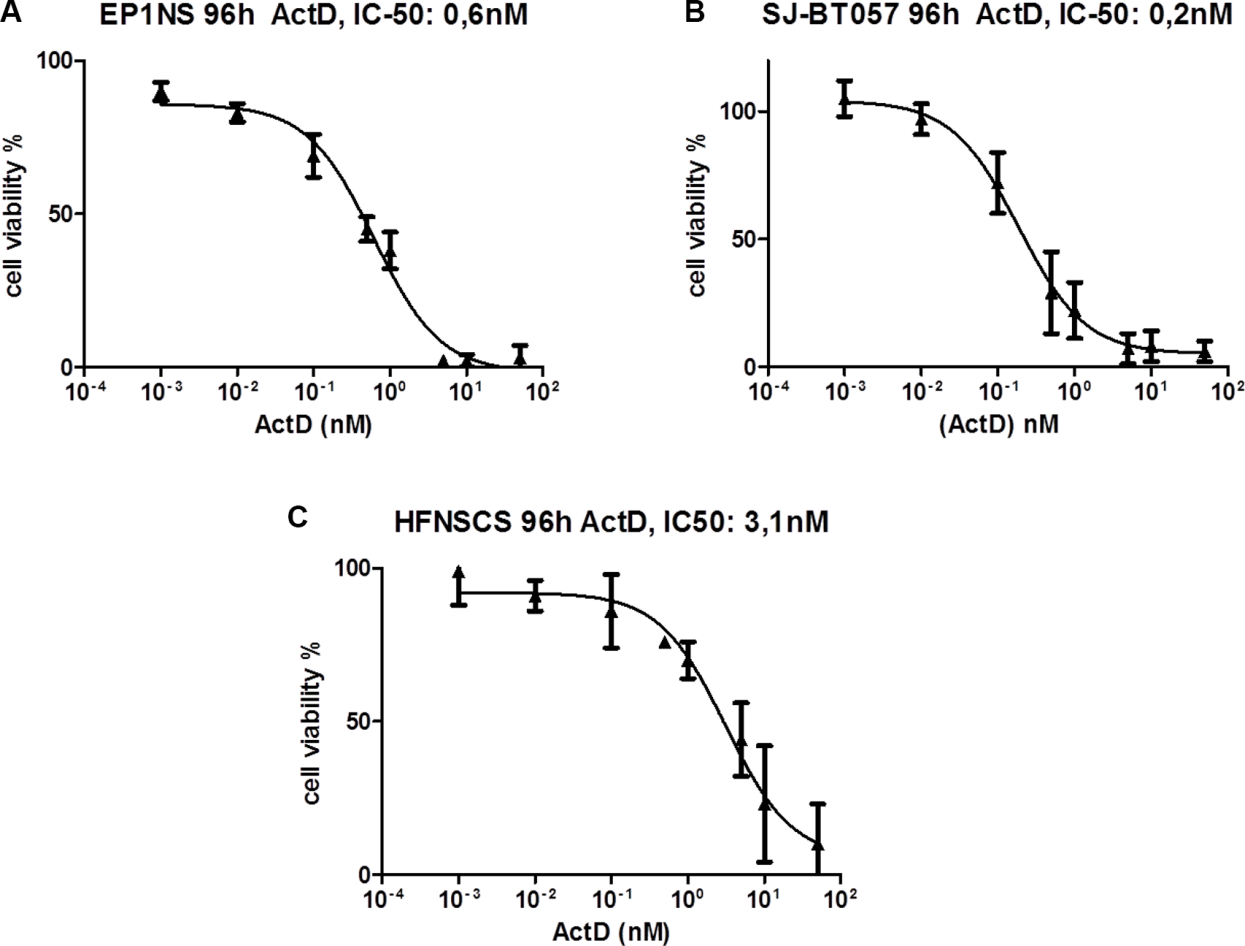
(**A**) MTS assay of EP1NS (ST-EPN-RELA) and (**B**) SJ-BT57 (PF-EPN-A) ependymoma cell lines, as well as (**C**) HFNSC neural stem cells after 96hours of treatment with different concentrations of Actinomycin-D showing a low-dose IC-50 in ependymomas and a 5-15 fold higher IC-50 in neural stem cells. IC, inhibitory concentration; Act-D, Actinomycin-D.

**Figure 3:**
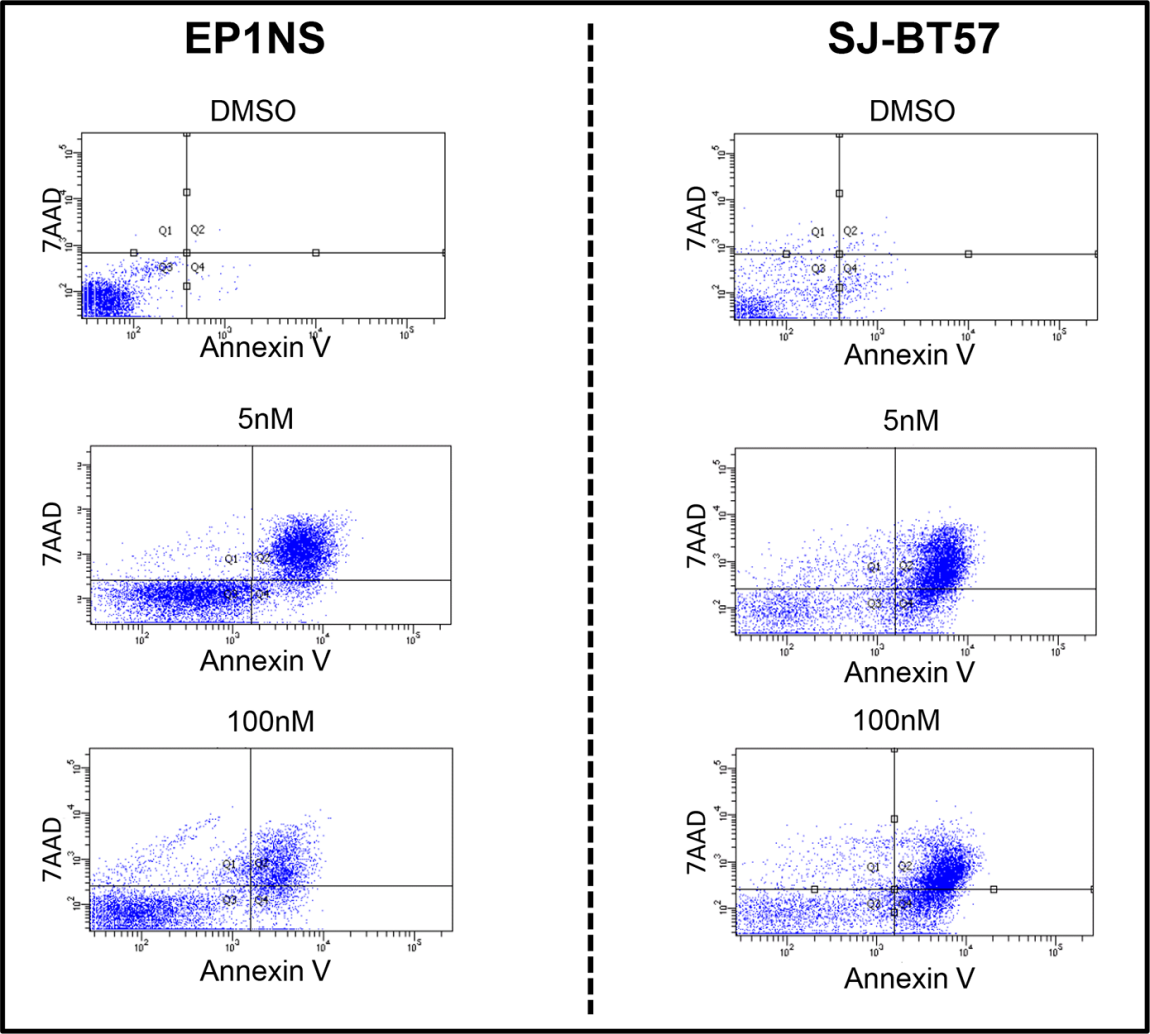
In the FACS analysis, low-dose (5 nM) treatment of the EP1NS cells proved to be more effective in apoptosis (44%, SD: +/−3%) induction than high-dose (100 nM; 21%, SD: +/−6%) application of the agent Actinomycin-D (left panel), whereas the SJ-BT57 cells showed a similar response to both doses (5 nM; 50%, SD +/−3%), (100 nM; 45%, SD +/−4%) (right panel) SD, standard deviation; 7AAD, 7-Amino Actinomycin-D.

Treatment of ependymoma cells with Actinomycin-D strongly inhibited their metabolic activity. The IC-50 of Actinomycin-D as measured by metabolic activity assay was in the low nano-molar range, i.e. 0.6 nM for EP1NS and 0.2 nM for SJ-BT57 (Figure 2A and 2B). The metabolic activity of human fetal neural stem cells (HFNSCS) was also affected by Actinomycin-D, but with an IC50 around 5-15 fold higher compared with the tumour cells (3.1 nM, Figure 2C). Observation of cells after treatment with different concentrations demonstrates that the agent did not merely inhibit their metabolic activity, but also disrupted neurosphere formation (Supplementary Figure S3), which was confirmed by flow cytometric analyses (Figure 3). Notably, low-dose treatment induced apoptosis more effectively compared to high-dose treatment in EP1NS, in contrast to SJ-BT57 cells, which showed a similar response to both doses (Figure 3).

Actinomycin-D treatment of the adult glioblastoma cell line U373 as well as of the paediatric medulloblastoma cell lines DAOY and UW228-3, all known to harbour an inactivating *TP53* mutation, showed either no effect (U373) or an inhibition of cell viability only at much higher concentrations similar to that of the embryonic stem cells (DAOY: 3 nM; UW228-3: 3 nM) (Supplementary Figure S4). When comparing the IC-50 of the ependymoma cells on the one hand and the medulloblastoma and fetal neural stem cells on the other hand, the non-ependymoma cells appear to need a 5–15 fold higher concentration of the agent to reach the same anti-proliferative effect.

The ependymoma cell lines were also treated with Nutlin-3, a small-molecule inhibitor of MDM2 with a similar mode of action to Actinomycin-D. When studying the metabolic response upon Nutlin3 treatment, the EP1NS cells exhibited an IC-50 of 1.2 μM (Supplementary Figure S5). In contrast, the viability of the SJ-BT57 cells appeared to be affected only by higher doses of the inhibitor showing a 8.3-fold higher IC-50 of 10 μM.

### Actinomycin-D and Nutlin-3 treatment of Group A and *RELA*-fusion positive ependymoma models *in vitro* induces apoptosis

Treatment of EP1NS cells with low-dose Actinomycin-D (5 nM) resulted in apoptosis in 44% of cells, while high-dose Actinomycin-D (100 nM) affected 21% of the cells (Figure 3). Similarly, the SJ-BT57 cells also responded to low-dose treatment (0.1 nM-5 nM) (Supplementary Figure S6). In this cell line, low concentrations of Actinomycin-D (5 nM) also induced apoptosis in 50% of cells, whereas similar, but not greater effects were observed after high-dose treatment (100 nM) with 45% of apoptotic cells (Figure 3). Apoptosis upon Actinomycin-D treatment of DAOY medulloblastoma cells was also determined in order to pinpoint the effects on a *TP53* mutated cell line. Interestingly, high-dose treatment with 100 nM resulted in apoptosis in 73% of DAOY cells compared to only 23% of apoptotic cells following treatment with 5 nM (Supplementary Figure S7).

Treatment of ependymoma cells with Nutlin-3 led to induction of early apoptosis in 12% by 100 nM, while 1 μM treatment of cells caused early complete apoptosis in nearly all of the EP1NS cells (Supplementary Figure S8). Treatment of the SJ-BT57 cells using 100 nM Nutlin-3 only induced apoptosis in 7% of cells, whereas 1 μM treatment led to 50% cell death.

### Actinomycin-D treatment reveals re-expression of p53-mediated pathway members on transcript and protein levels

**Figure 4:**
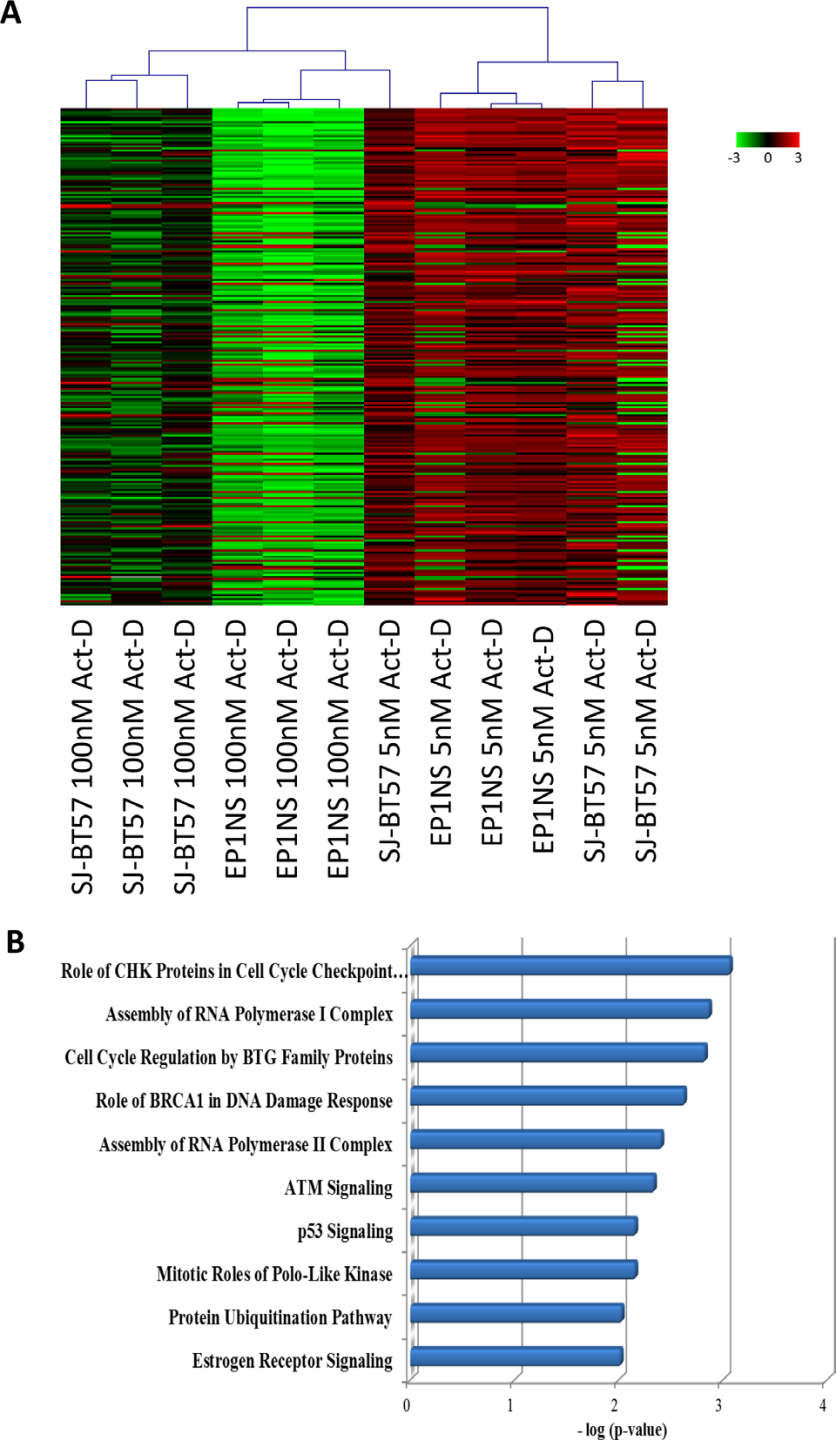
(**A**) Gene expression profiling after high-dose (100 nM) and low-dose (5 nM) Actinomycin-D treatment of ependymoma cell lines, EP1NS (ST-EPN-RELA) and SJ-BT57 (PF-EPN-A). Unsupervised hierarchical clustering of top 300 differentially expressed genes revealed distinct transcriptome influences according to treatment. (**B**) Ingenuity pathway analysis of significant differentially regulated genes between high-dose (100 nM) and low-dose (5 nM) treatment of the cell lines pinpointed the p53 pathway as one of the top ten upregulated pathways after low-dose treatment.

Unsupervised clustering of transcriptomic data following high-dose (100 nM) and low-dose (5 nM) Actinomycin-D treatment of ependymoma cell lines EP1NS and SJ-BT57 revealed two distinct clusters, with samples being separated according to the respective treatment intensity rather than their subgroup affiliation (Figure 4A). Pathway analysis based on gene expression data identified members of CHK proteins involved in the cell cycle checkpoint control as the top affected pathway with highest level of significance (*p* = 0.0009; Figure 4B). These associated genes were upregulated after low-dose and downregulated after high-dose Actinomycin-D. Among the top differentially regulated pathways were several apoptosis and cell-cycle arrest-associated signalling cycles such as cell cycle regulation by BTG proteins (*p* = 0.001), BRCA1 role in DNA damage response (*p* = 0.002), ATM signalling (*p* = 0.005), the protein ubiquitination pathway (*p* = 0.009) and the P53 pathway (*p* = 0.007), all of which were upregulated after low-dose and downregulated after high-dose Actinomycin-D treatment (Figure 4B).

**Figure 5:**
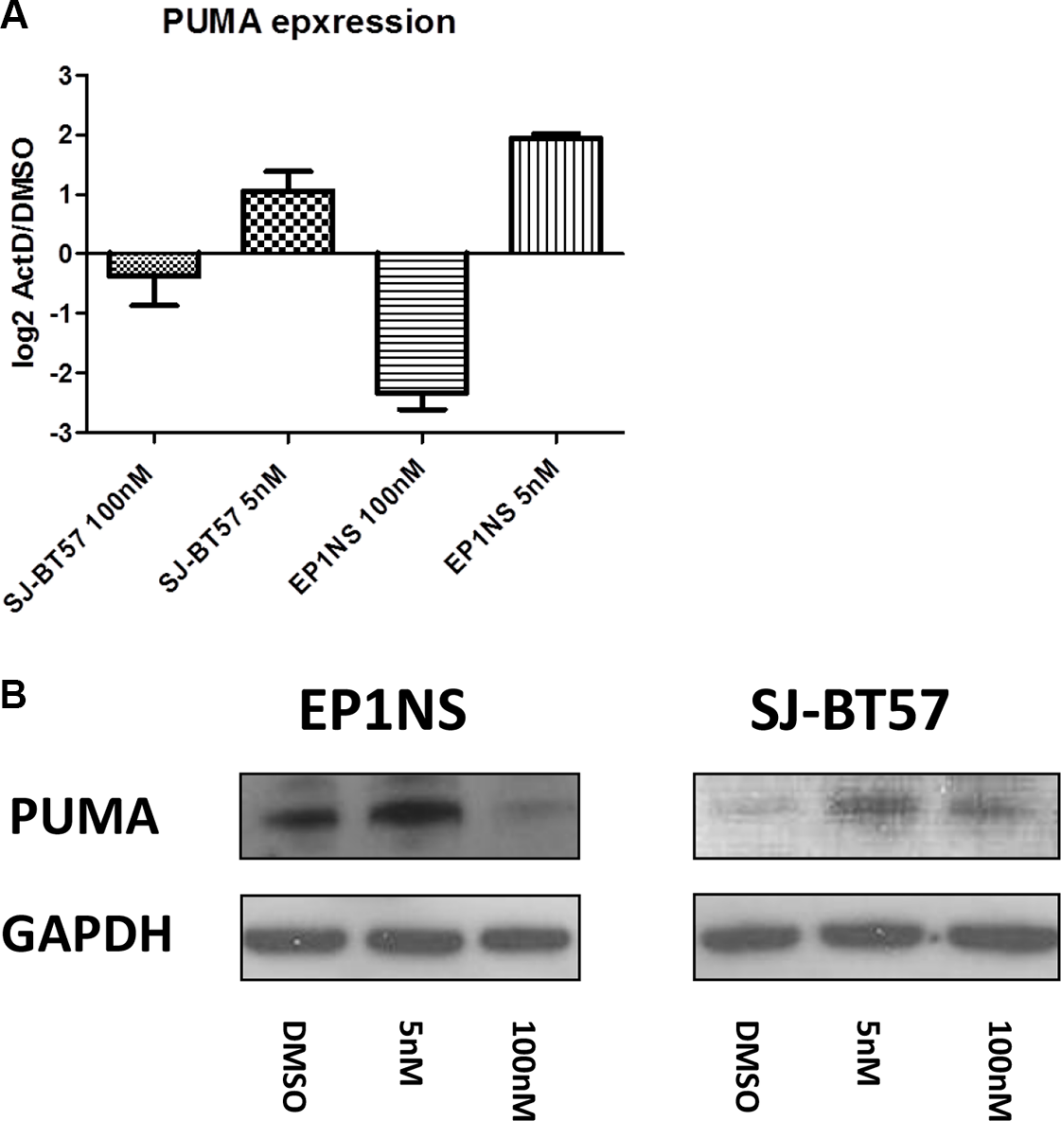
A high proportion of p53 dependent genes such as PUMA were found to be upregulated after low-dose (5 nM) and downregulated after high-dose (100 nM) Actinomycin-D treatment of both cell lines This was proven at RNA (**A**) and protein (**B**) level. GAPDH, Glycerinaldehyd-3-phosphat-Dehydrogenase.

Interestingly, when performing separate pathway analyses for the two different ependymoma cell lines, the pathway of differentially expressed genes bearing the highest level of significance was the p53 pathway in the *RELA*-positive EP1NS cells (ST-EPN-RELA), whereas for the SJ-BT57 cells (PF-EPN-A), it was the 10th most significant differentially regulated pathway (*p* = 0.01; Supplementary Figure S9). Among the various genes with altered expression as a result of Actinomycin-D treatment, one of the most differentially expressed genes was *p53 upregulated modulator of apoptosis* (*PUMA*), also known as *Bcl*-2-binding component 3 (*BBC3*), a known p53 target gene promoting apoptosis. Interestingly, PUMA is involved in p53 regulation and p53-mediated apoptosis. Thus, we aimed to validate PUMA regulation on protein level (Supplementary Table S2). After Actinomycin-D treatment *in vitro* of preclinical ependymoma models (EP1NS as ST-EPN-RELA and SJ-BT57 as PF-EPN-A) PUMA was also shown to be upregulated on both the mRNA (Figure 5A) and protein level, when compared to matched loading controls (Figure 5B).

**Figure 6:**
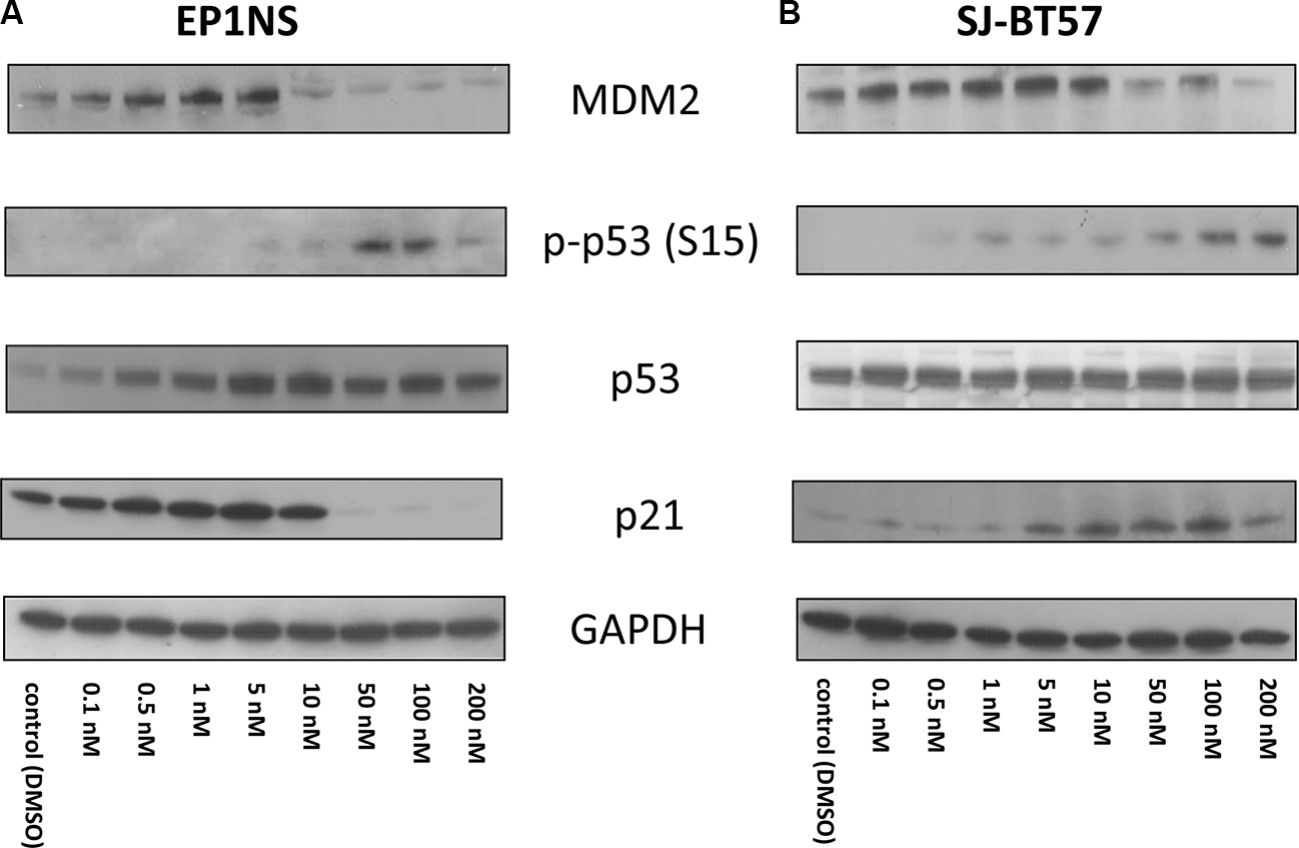
(**A**) For the EP1NS cells (ST-EPN-RELA), high- and low-dose Actinomycin-D treatment caused an overexpression of p53, however both p21 and MDM2 were maximally induced after 5 nM treatment and downregulated after high-dose Actinomycin-D confirming a more effective p53 reactivation after low-dose treatment. (**B**) The SJ-BT57 cells (PF-EPN-A) demonstrated no changes at p53 expression, but a p21 induction after low- and high-dose treatment and a MDM2 activation only after low-dose treatment proving mainly low-dose Actinomycin-D to be capable of re-establishing the p53 complex.

After Actinomycin-D treatment of EP1NS cells, p53 induction was detected using western blotting, which reached a plateau at 10 nM. Interestingly, high levels of phosphorylated p53 at Serine 15, a supposed indicator of the functional form of the protein [34], were detected after 10 nM and high-dose treatment (Figure 6A). This might be seemingly contradictory, yet it has already been proposed that phosphorylation of p53 does not play an important role in p53 stabilisation [35] and is not always required for the transcriptional function of p53 and apoptosis induction [36]. For p21, one of the most important target genes of p53, low-dose concentrations of the agent (10 nM, 5 nM, 1 nM, 0.5 nM) induced a high expression of the protein in EP1NS cells, whereas it was hardly detectable after 50 nM, 100 nM and 200 nM treatment (Figure 6A). Active p53 is known to be upregulating MDM2, the expression levels of which increased dramatically after low-dose Actinomycin-D treatment (5 nM, 1 nM and 0.5 nM). After treatment with 10 nM and highdose application of the agent, MDM2 expression was downregulated (Figure 6A).

Actinomycin-D treatment of SJ-BT57 cells exhibited a different pattern (Figure 6B). Only minor changes in total p53 were observed, whereas phosphorylated p53 at Serine 15 site accumulated after high-dose treatment. Induction of p21 was observed after low-dose as well as after high-dose application of the agent mainly after treatment with 5 nM, 10 nM, 50 nM, 100 nM and 200 nM, with an induction peak at 100 nM. Finally, MDM2 overexpression was detected only after low-dose Actinomycin-D treatment with 10 nM, 5 nM, and 1 nM (Figure 6B).

On the contrary, DAOY cells did not exhibit any difference in expression of p21, p53 and MDM2 regardless of the concentration of Actinomycin-D (Supplementary Figure S10). Notably, a p53 and MDM2 protein accumulation was observed, whereas p21 showed a low expression throughout all the concentrations of Actinomycin-D and the DMSO control.

To monitor the dynamics of p53 induction, Western Blot experiments were performed after 2 hours, 6 hours and 8 hours of treatment with Actinomycin-D applied (Supplementary Figure S11). EP1NS cells demonstrated a progressive increase of p53, MDM2 as well as p21 after treatment with 5 nM, whereas the DMSO control counterparts did not show any changes in the protein levels of these molecules. Changes in SJ-BT57 cells were also detected, with p21 and MDM2 showing a progressive increase after 5 nM treatment compared to the unaltered expression of the products at the DMSO control. Expression of p53 in this line was equally high in both cell lines, with no differences noted between 5 nM and DMSO control or in time course (Supplementary Figure S11).

When assessing the resulting protein modifications after Nutlin-3 treatment of the ependymoma cell lines, the findings differed greatly between the cell lines (Supplementary Figure S12). EP1NS cells showed dose-dependent elevation of p53 and p53-dependent targets p21 and MDM2. Notably, p53 was highly increased after 10 μM, 20 μM and 40 μM treatment, whereas p21 and MDM2 induction reached a plateau at 10μM treatment. On the contrary, the SJ-BT57 cells did not demonstrate any differences in p53, p21 and MDM2 expression after Nutlin-3 treatment (Supplementary Figure S12).

**Figure 7:**
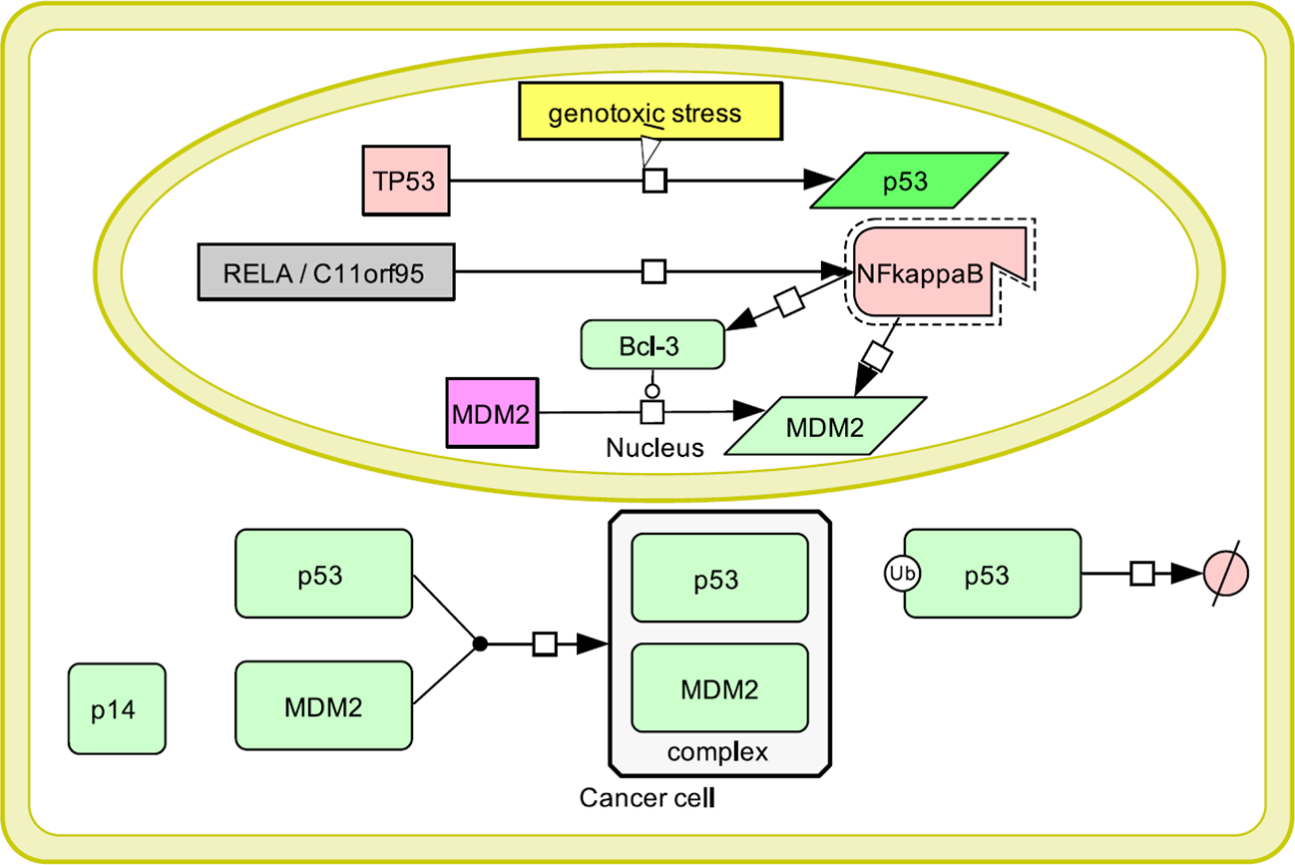
Model of p53 inactivation in ependymoma: Genomic instability and thus genotoxic stress in a cancer cell induces p53 expression, which then allocates to the cytoplasm *RELA-C11orf95* fusion leading to constitutive NF-kappaB activation results into MDM2 activation directly or through transcriptional upregulation of NF-kappaB transcriptional target Bcl-3. MDM2 allocates to the cytoplasm, where it forms a complex with p53 leading to its degradation. An additional mechanism of MDM2 upregulation includes absence of p14-mediated inhibition due to homozygous *CDKN2A* deletion occasionally observed in high-risk ependymomas. Thus, p53-mediated apoptosis does not occur and cancer cells continue to proliferate.

As previously mentioned, MDM2 was shown to be induced by aberrant NF-kappaB activation. Different mechanisms proposed in embryonic fibroblasts and breast carcinoma cells included transcriptional activation of MDM2 induced by NF-kappaB transcriptional targets [37, 38]. Thus, p53 inactivation could also be associated with NF-kappaB upregulation in *RELA*-positive ependymomas. Our scheme (Figure 7) demonstrates a proposed model for a possible mechanism of functional impairment of p53 in ependymoma, although this pathway still has the potential to be further explored.

## DISCUSSION

The evident lack of targeted therapeutic options for high-risk ependymomas, i.e. ST-EPN-RELA and PF-EPN-A (Group A), represents a major drawback in clinical practice. In this study, MDM2 and p53 were identified as potential drug targets in ST-EPN-RELA ependymoma *in vitro*, with low-dose Actinomycin-D being capable of reactivating p53 function and thereby re-establishing its tumour-suppressive role, whereas Nutlin-3 mediated similar effects yet only in the ependymoma cell line representing a supratentorial *RELA*-positive ependymoma subtype harbouring an additional homozygous *CDKN2A* deletion.

The tumour suppressor gene *TP53* is rarely mutated, but frequently inactivated in ependymomas, as evidenced by p53 accumulation (particularly in ST-EPN-RELA) and its correlation with worse prognosis. A possible mechanism of p53 inactivation could involve NF-kappaB upregulation in *RELA*-positive ependymomas. The interaction of p65 as a downstream target of the NF-kappaB signalling pathway and p53 causes tumour development and tumour progression [39]. *MDM2* can be regulated transcriptionally by both NF-kappaB and p53, and was itself found to regulate the NF-kappaB pathway through binding and inhibition of the p65 subunit [40].

In particular, MDM2 has been demonstrated to be induced by NF-kappaB signalling thereby resulting in p53 inactivation [16]. A plausible mechanism involving NF-kappaB transcriptional target Bcl3 as an oncogene suppressing p53-dependent apoptosis due to MDM2 activation has been described in breast carcinoma cells [37]. Another approach using mouse embryonic fibroblasts revealed IKK2, member of the NF-kappaB complex, as a mediator of p53 silencing due to MDM2 upregulation thereby offering a possible explanation for resistance to conventional chemotherapy [38]. Future functional *in vitro* and *in vivo* studies are warranted to further investigate the cross-talk between NF-kappaB associated genes and transcriptional regulators and p53 within *RELA*-positive ependymomas.

Since a smaller subset of high-risk tumours exhibit a homozygous *CKDN2A* deletion leading to absence of p14 and thereby excessive MDM2 activity, this could comprise an alternative mechanism of p53 inactivation in these tumours [41]. This phenomenon was observed only in intracranial ependymomas in our data set [11] and in our cell line EP1NS harbouring a defective p53 pathway. These two events of p53 accumulation and abnormal activity of MDM2 appear to be mutually exclusive, since MDM2 promotes p53 degradation, thereby contradicting the high levels of immunohistochemically detected p53 [42]. However, it has previously been suggested that NF-kB activation leads to accumulation of non-functional p53 in breast carcinoma cells. [43] Treatment of these cells with TNFα leading to aberrant NF-kB activity showed elevated p53 levels, yet without an upregulation of p53-linked transcriptional targets or induction of p53-associated apoptosis. In another study, NF-kB was shown to be upregulated in tumour cells leading to p53 inactivation via endogenous glucocorticoids and thus to tumour progression [44].

Surprisingly, phosphorylation of p53 at the Serine 15 site was detected in both cell lines after highdose Actinomycin-D and not at all after low-dose treatment, in contrast to the findings of Choong and colleagues [30]. However, this subject has always been controversial, since it has already been proposed that phosphorylation of p53 does not play an essential role in p53 stabilisation [35] and is not always required for the transcriptional function of p53 and apoptosis induction [36]. Interestingly, treatment with Actinomycin-D and Nutlin-3 exhibited distinct differences between the two cell lines. While Actinomycin-D induced late-apoptosis, Nutlin-3 seemed to induce early apoptosis after cell treatment for 48 hours. It is known that externalisation of phosphatidil-serine to the outer cell membrane layer mediating a phagocytic signal occurs at an early apoptotic phase and is detected by Annexin V, while late apoptosis or necrosis leads to exposure of cationic molecules such as 7AAD [45]. A possible explanation for this phenomenon would include a delay in Nutlin-3 mediated apoptosis due to pharmacokinetic profile. Since 7AAD/Annexin V positive cells could also represent a subset of necrotic cells, one could suppose that Nutlin-3 is better tolerated than the cytostatic drug Actinomycin-D.

Moreover, according to Rao et al. [46], different responses to Actinomycin-D and Nutlin-3 might be explained through molecular differences and pharmacokinetics of the compounds, yet they observed that Nutlin-3 caused a p53 reactivation much more rapidly than Actinomycin-D due to the molecular mechanism of action. The lack of effects in SJ-BT57 cells could be attributed to the much higher p53 accumulation in the untreated cells, rendering the agent incapable of unfolding its action through MDM2 inhibition. The higher MDM2 levels of untreated SJ-BT57 cells compared to the EP1NS cells could also support this argument, concluding that p53 accumulation could possibly account for MDM2 expression as a negative feedback mechanism and thus prevent Nutlin-3 from sufficiently inhibiting MDM2 due to already existing higher MDM2 levels. The observed apoptosis could thus comprise a non-specific, p53-independent response to the drug.

Future studies to further assess the usefulness of Actinomycin-D for *RELA*-positive ependymoma, should include *in vivo* preclinical experiments combined with pharmacokinetic and pharmacodynamic measurements to improve the understanding of therapeutic drug concentrations in the serum and CSF. However, the reported peak plasma drug concentration of Actinomycin-D is 25,1 ng/mL equalling 20 nM (Veal et al., 2005), thus about two orders of magnitude higher than the IC 50 values found in the cell line experiments (0,6 and 0,2 nM) thereby implicating that the suggested dosage of Actinomycin-D comprises clinically achievable concentrations. Intraperitoneal treatment of mice with Actinomycin-D has previously been conducted in studies concerning Wilms tumour and prostate cancer [47, 48]. Nutlin-3 has also been tested *in vivo* via either oral or intraperitoneal application in various tumour models [49–51]. Hence, Actinomycin-D and Nutlin-3 treatment via intraperitoneal and/or oral application in mice with orthotopically implanted ependymoma cells could provide a useful setting for preclinical drug testing in future *in vivo* studies. Despite reports showing limited or even absent effects of chemotherapy in pediatric ependymomas [3], a combination therapy with conventional multialkylating chemotherapy could be considered. Yet, Actinomycin-D comprises a clinically more feasible treatment option than conventional chemotherapy due to the absence of severe secondary effects.

Thus, there is an urgent need for several distinct reliable and well-established preclinical ST-ENP-RELA and PF-ENP-A *in vivo* models in mice to further test Actinomycin-D and Nutlin-3 in ependymoma. Herewith, a confirmation of the re-establishment of p53 function *in vivo* can pave the way for a clinical application for patients suffering from high-risk ependymoma with frequent p53 silencing.

In summary, we were able to prove that low-dose Actinomycin-D and Nutlin-3 are capable of re-establishing the tumour-suppressive function of p53 in two preclinical ependymoma models *in vitro*. This could possibly implicate a rationale for a clinically feasible treatment option for ST-EPN-RELA ependymoma patients with low-dose Actinomycin-D.

## MATERIALS AND METHODS

### Cell culture

DKFZ-EP1NS [25], SJ-BT57 [52], and human fetal neural stem cells obtained from C. Watts, University of Cambridge were cultivated as neural stem cells using neurosphere medium (Invitrogen, Karlsruhe, Germany) with growth factor supplements as described [25] Medulloblastoma cells DAOY (obtained from American Type Culture Collection) and UW228-3 cells (obtained from Steven Clifford, Newcastle, United Kingdom), were cultivated adherently using normal DMEM (Dubecco's modified Eagle medium, Invitrogen, Karlsruhe, Germany). Based on DNA methylation profiling using Illumina 450k methylation array [11], cell lines EP1NS and SJ-BT57 were classified as preclinical *RELA*-positive (EP1NS) and posterior fossa Group A (SJ-BT57) models. Additionally, according to the study of Parker and colleagues [15], the *RELA-C11orf95* fusions was detected in EP1NS by reverse-transcription PCR, detailed description can be found elsewhere [11, 15].

### Metabolic activity assays

Cell viability was observed by measuring metabolic activity of the cells (EP1NS, SJBT57, HFNSCS, DAOY, UW228-3 and U373) after treatment with different concentrations of Actinomycin-D and Nutlin-3 (both by Sigma-Aldrich, Hamburg, Germany) was assessed by MTS based CellTiter96^®^ AQ Non-Radioactive Cell Proliferation Assay (Promega, Madison, USA). After seeding of the cells in flat-bottom 96-well plates at a density of 5000 cells/well and treatment with Actinomycin-D for 96 hours, the MTS reagent was applied. The subsequent incubation time was 3 hours, the signal intensities were measured by an EL800 Universal Microplate Reader (BIO-TEK Instruments Inc., Winooski, USA). Half-maximal inhibitory concentrations (IC50) were calculated using GraphPad Prism version 5.0 (Graph Pad Software, La Jolla, CA, USA).

### Flow cytometry

Flow cytometrie (FACS) was performed as follows. After cell treatment with high and low-dose Actinomycin-D and Nutlin-3 for 48 hours, cells were washed with PBS, followed by digestion and mechanical dissociation, as previously described. Enzymatic reaction was stopped with FACS-buffer (10% fetal calf serum solved in PBS). Subsequently, cells were stained with 7-Actinomycin-D and Annexin V (BD Pharmingen, Heidelberg, Germany), both in a 1:10 dilution (7-AAD, Annexin V, FACS buffer) for 20 minutes at 4°C. Staining reaction was stopped with annexin buffer and for the flow cytometric analysis a BD FACS Canto II machine was applied. For analysis, the software FACSDiva (version 6.1.2; Beckton Dickinson) was used.

### TP53 sequencing

DNA was isolated according to the manufacturer's instructions using the blood and cell culture kit (Qiagen, Hilden, Germany). Polymerase Chain Reaction (PCR) was carried out to amplify the different *TP53* exons (2–11) as shown previously [53]. Thereafter, agarose gel electrophoresis was performed, in order to confirm the amplification of each *TP53* exon. PCR was carried out using the ABI PRISM Big Dye terminator cycle sequencing ready reaction kit (Applied Biosystems Inc, Foster City, USA). Sequencing and data collection were performed on the ABI PRISM 3100 Genetic Analyser, software: version 1.6 (Applied Biosystems Inc, Foster City, USA).

### Gene expression profiling

Cells were treated for 6 hours with high-dose (100 nM) and low-dose (5 nM) Actinomycin-D, or DMSO as a control was included. RNA extraction was performed using a RNeasy Mini Kit (Qiagen). For quality control check, Agilent Bioanalyzer RNA nano-chips (Agilent, Santa-Clara, CA, USA) were used. An Agilent 44 K array (Agilent, Santa Clara, CA, USA) transcriptome analysis was conducted following the manufacturer's instructions. The two colour hybridization was performed using RNA from Actinomycin-D treated cells versus RNA of DMSO treated cells (control). The reaction was carried out for 17 hours and the slides were subsequently scanned using the Agilent DNA Microarray Scanner Model G2505B. The microarray data were extracted and pre-processed using the Agilent Feature Extraction Software, followed by incorporation into the microarray data analysis software ChipYard (http://www.dkfz.de/genetics/ChipYard/) as previously described [54].

Multi Experiment Viewer (MeV) software (J. Craig Venture Institute, La Jolla, USA) was used to analyse the data [55]. Unsupervised hierarchical clustering was performed, in order to assess the significance analysis of microarrays (SAM) including a number of 20 permutations and a false positive rate of 1% were applied, to distinguish the significant differentially expressed genes. The data were loaded to the Ingenuity pathway analysis platform, to distinguish the differentially expressed molecular pathways. Data were analysed through the use of QIAGEN's Ingenuity^®^ Pathway Analysis (IPA^®^, QIAGEN Redwood City, www.qiagen.com/ingenuity). Top 10 significant differentially expressed pathways, as assessed by Ingenuity Pathway Analysis, were analysed.

### Western blotting

Cells were treated for 6 hours with Actinomycin-D and lysis was performed using RIPA (Radioimmunoassay precipitation) buffer. Protein concentration was measured with a Qubit 2.0 Fluorometer (Invitrogen, Karlsruhe, Germany). For electrophoresis at 150 V for 90 minutes, 10 μg of the protein were loaded on a 4–20% Tris-Bis gradient gel (Invitrogen). For blotting a PVDF membrane (Immobilon, Merck Millipore, Darmstadt, Germany) was used. After exposure to the secondary antibody for 1 hour at room temperature, Amersham ECL Advance Western blotting detection system (GE Healthcare, Buckinghamshire, UK) was applied to perform a conventional film development. Antibodies against p53 (BD Pharmingen, mouse, 1:1000), p21 (BD Pharmingen, mouse, 1:200), MDM2 (Sigma, mouse, 1:500), PUMA (Abcam, rabbit, 1:500), p14 (Abcam, mouse, 1:500), and GAPDH (Cell Signalling, mouse, 1:5000) were used in the above-mentioned concentrations.

### Immunohistochemistry

Ependymoma tissue microarrays (TMA) were prepared using 406 paraffin blocks of ependymoma samples, in addition to 10 samples of non-tumour brain tissues were included as a control (Witt et al., 2011). Ependymoma samples of the tissue microarray were collected from a single-centre study at the Burdenko Neurosurgical Institute in Moscow, Russia. All tumours were banked at the time of primary diagnosis between 1993 and 2007, and diagnosed as M0 at the time of surgical resection. At least 80% of tumour cell content was estimated in all TMA samples by staining cryosections (~5 μm thick) of each sample with hematoxylin and eosin. Diagnoses were confirmed by histopathologic assessment by at least two neuropathologists, including a central pathology review that utilized the 2007 WHO classification for CNS tumours. Details of this cohort are shown previously (Witt et al., 2011). An antibody against the following antigen was used: p53 (BD Pharmingen, mouse, 1:1000). TMA staining was performed, evaluated and scored as published. The cut-off scoring p53 positive cases is 10% of strongly stained nuclei, which is a well-established and widely used threshold level in neuropathology (Witt et al., 2011).

### Statistical analysis

Statistical significance for immunohistochemical analysis on the tissue microarray was examined using a non-parametric *log-rank test*.

For gene expression profiling, significance analysis of microarrays (SAM) including a number of 20 permutations and a false positive rate of 1% (*q* ≤ 1%) were used as a cut-off, in order to distinguish the significant differentially expressed genes.

In the platform of Ingenuity pathway analysis (IPA^®^, QIAGEN Redwood City, www.qiagen.com/ingenuity), a right-tailed Fisher Exact test is used to highlight significant differentially regulated pathways.

## SUPPLEMENTARY MATERIALS FIGURE AND TABLES


