# Supplementary material for: Low-dose Actinomycin-D treatment re-establishes the tumoursuppressive function of P53 in RELA-positive ependymoma
Source: Oncotarget. 2016 Aug 20;7(38):61860–73. doi: 10.18632/oncotarget.11452 (PMC5308696; doi:10.18632/oncotarget.11452)
Supplement: Supplementary file 1 [file oncotarget-07-61860-s001.pdf]

Low-dose Actinomycin-D treatment re-establishes the tumour-suppressive function of P53 in *RELA*-positive ependymoma

Supplementary Materials

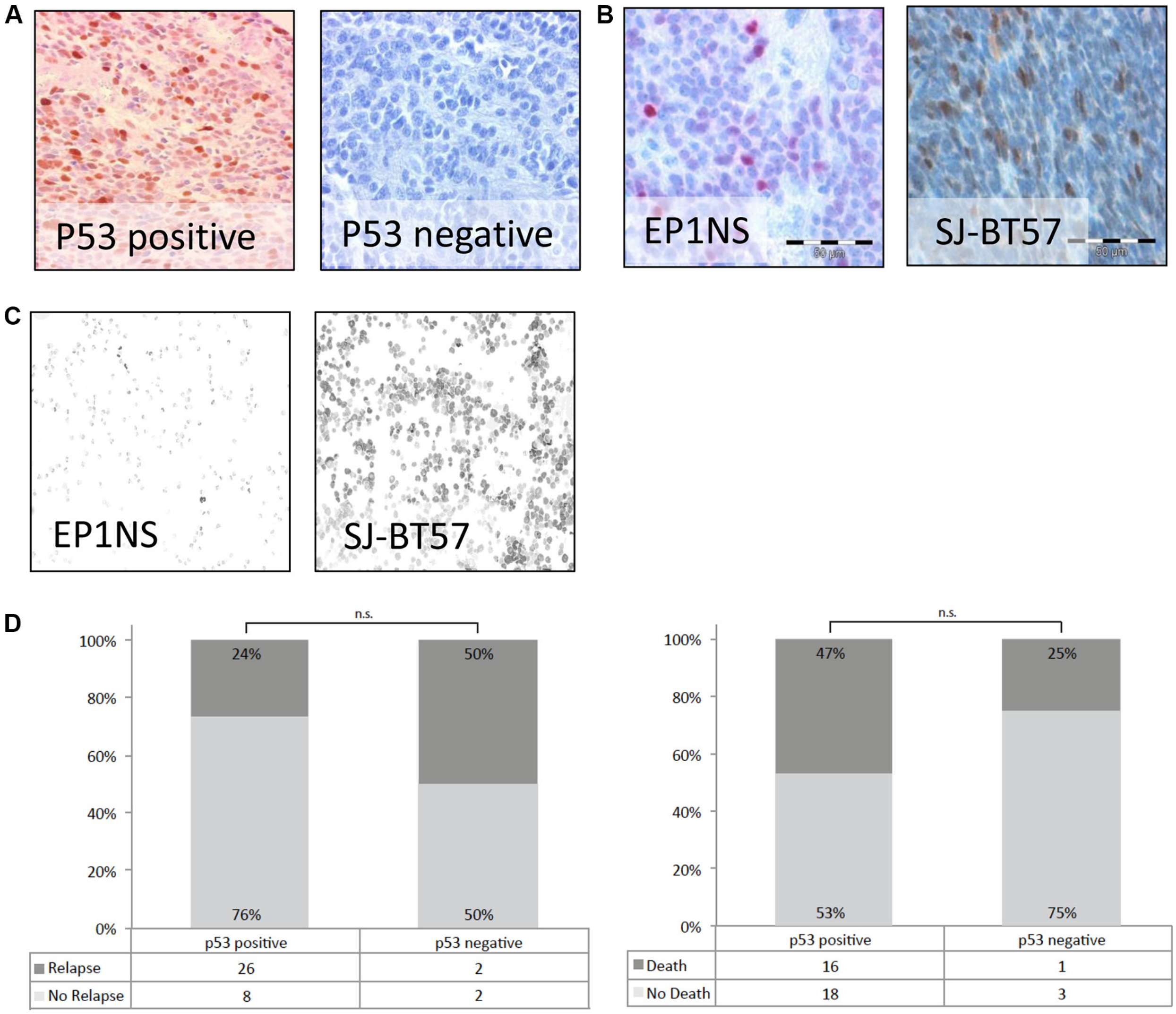

Supplementary Figure S1: (**A**) Representative pictures of a p53 positive and a p53 negative primary ependymoma after IHC staining. (**B**) Representative pictures of p53 IHC performed on mouse brain sections after orthotopical transplantation of EP1NS and SJ-BT57 cells in the posterior fossa. The EP1NS mouse tumours exhibit a moderate p53 staining, whereas SJ-BT57 tumours show a stronger p53 accumulation. (**C**) Immunohistochemistry of p53 *in vitro* of EP1NS (left) and SJ-BT57 (right) cells showing a strong p53 accumulation in SJ-BT57 and a moderate p53 signal in EP1NS cells. (**D**) Supratentorial *RELA*-positive ependymoma (n = 38; TMA cohort) show no significant association between p53 IHC positivity and relapse (left; $P = 0.29$) or mortality (right; $P = 0.61$) using *Fisher's exact test*.

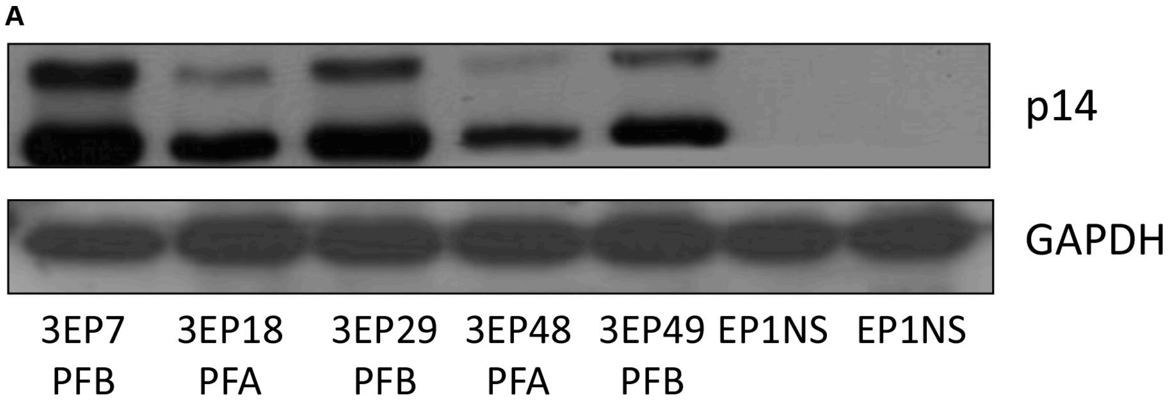

Supplementary Figure S2: Protein analysis of primary ependymomas (PF-EPN-A and PF-EPN-B) and two samples of the EP1NS cells (duplicate) showing the absence of p14 in the EP1NS cells underlining the homozygous *CDKN2A* loss in the EP1NS (ST-EPN-RELA) cell line.

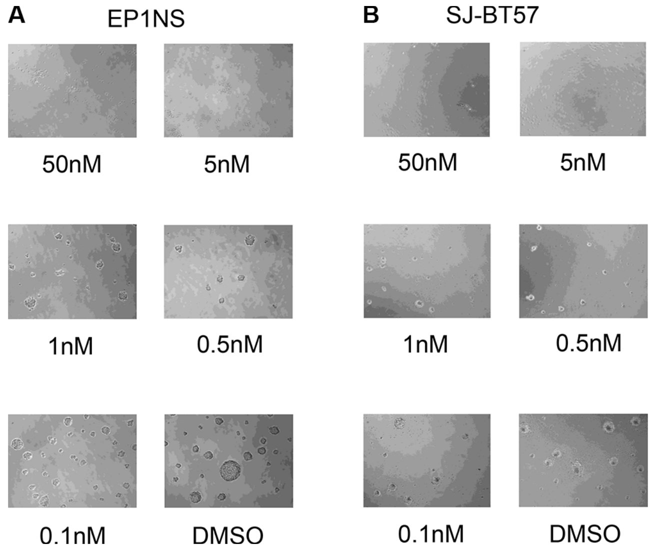

Supplementary Figure S3: Microscopic pictures of the EP1NS (A) and SJ-BT57 (B) cells after 96 hours Actinomycin-D treatment at different concentrations as labelled and the DMSO control showing the disruption of neurospheres after low- and high-dose treatment.

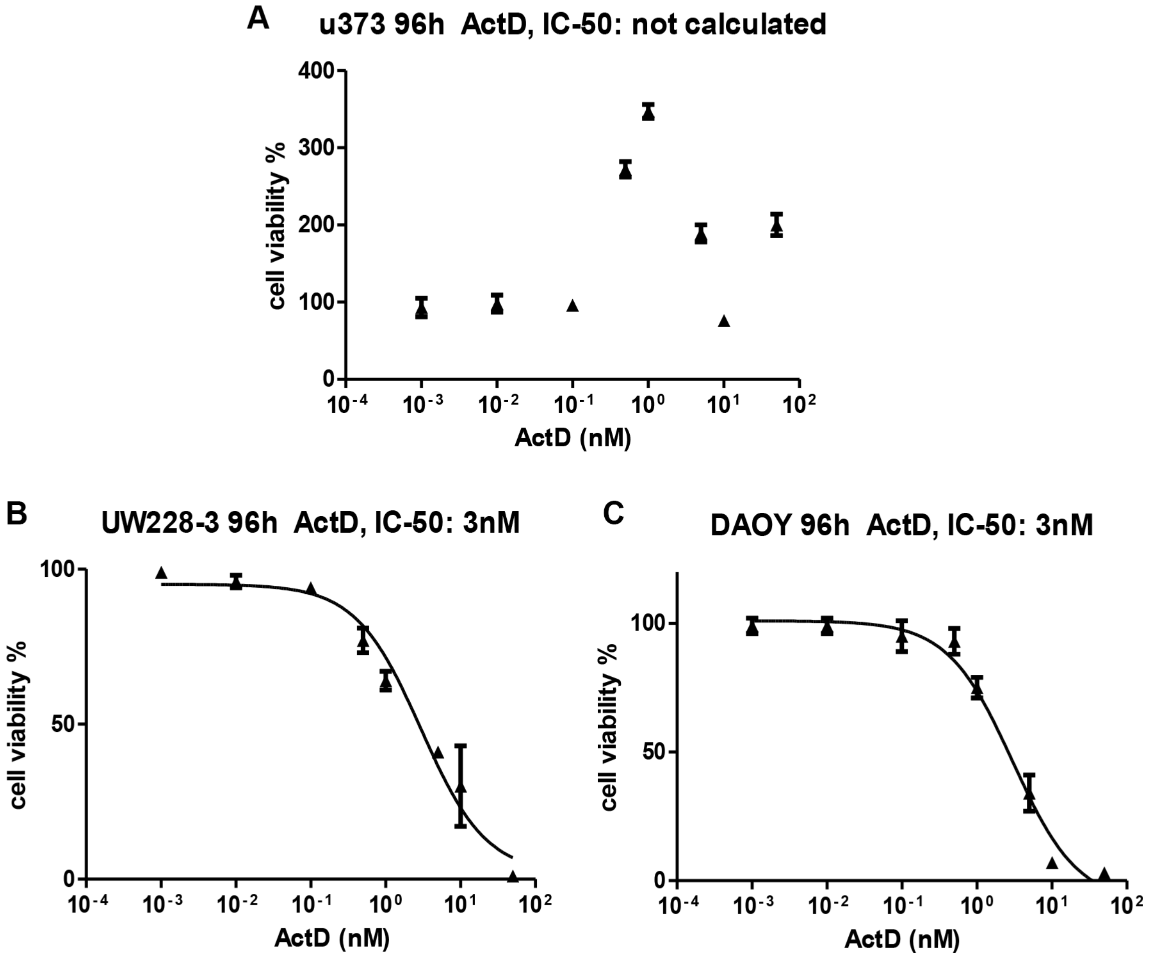

Supplementary Figure S4: MTS cell viability assay of 96 hours Actinomycin-D treatment of different cell lines of glioblastoma (A) and medulloblastoma (B, C) cells. ActD, Actinomycin-D; IC, Inhibitory Concentration.

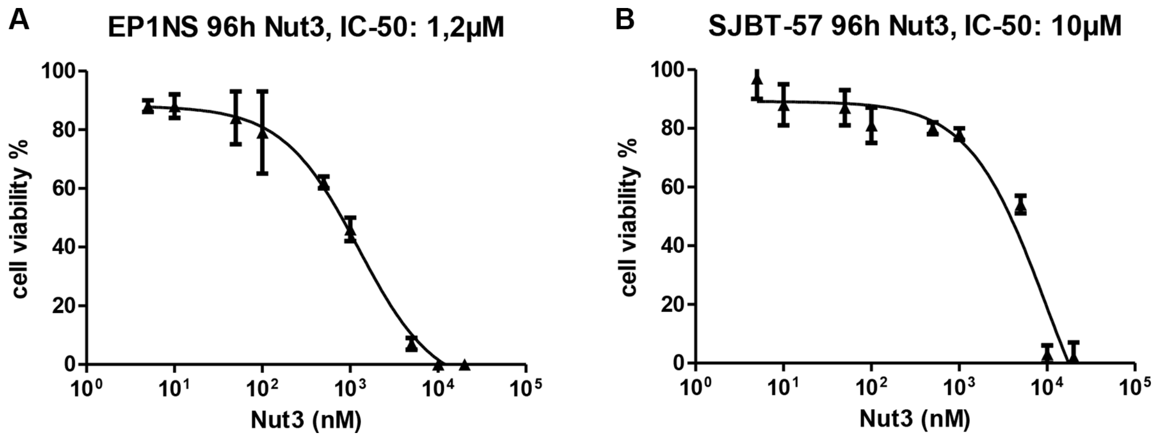

Supplementary Figure S5: MTS cell viability assay of EP1NS (A) and SJ-BT57 (B) ependymoma cell lines after 96 hours treatment with different concentrations of Nutlin-3 showing an IC-50 of 1.2 µM for the EP1NS and a 8.3-fold higher IC-50 for the SJ-BT57 cells.

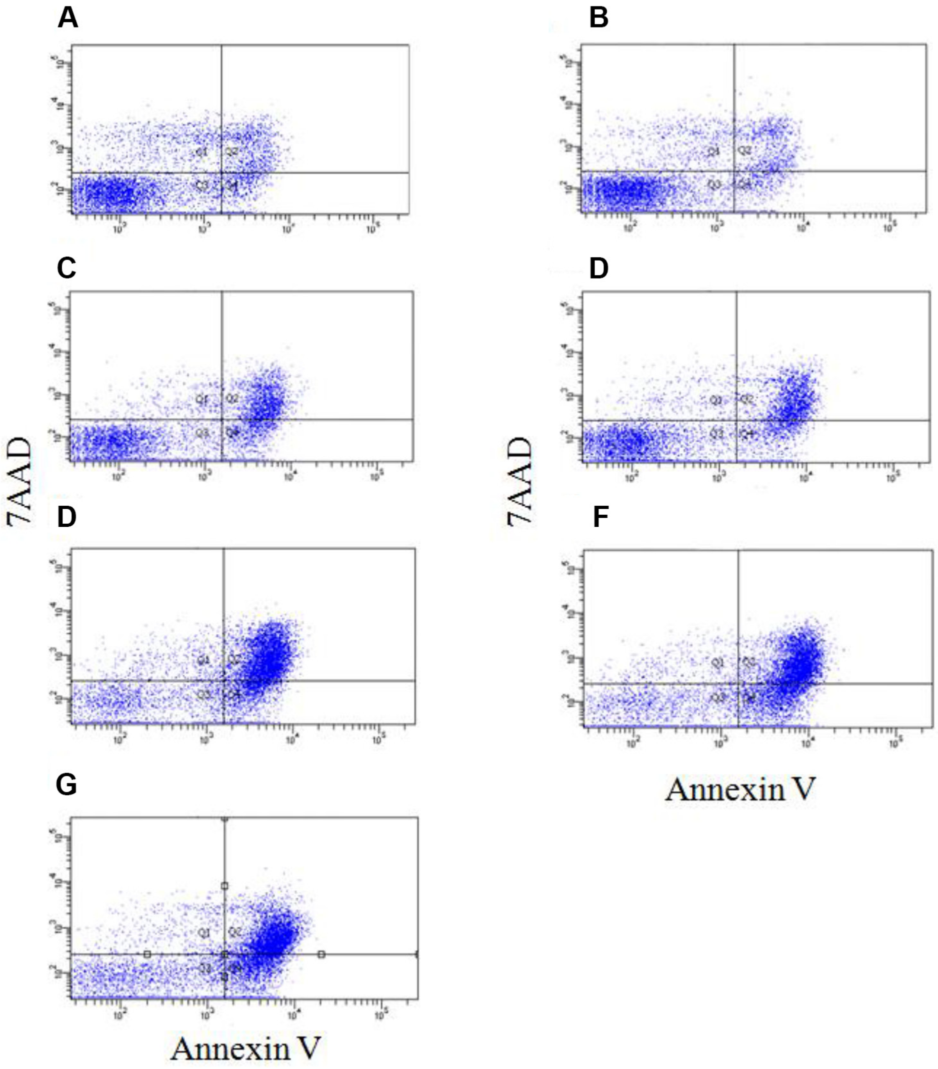

Supplementary Figure S6: Flow cytometric analysis with 7AAD and Annexin V staining of Actinomycin-D treated SJ-BT57 cells for 48 hours. The diagrams represent the DMSO control (**A**) as well as different concentrations of the agent including 0.1 nM (**B**), 0.5 nM (**C**), 1 nM (**D**), 5 nM (**E**), 10 nM (**F**) and 100 nM (**G**).

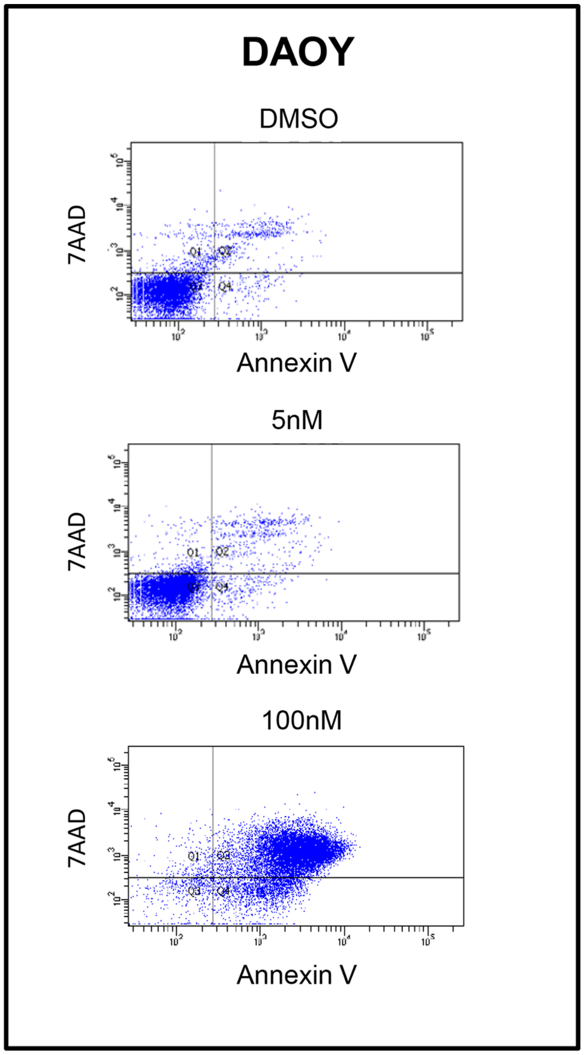

Supplementary Figure S7: FACS analysis of low-dose (5 nM) and high-dose treatment of the DAOY cells demonstrating apoptosis only after high-dose treatment of the cells.

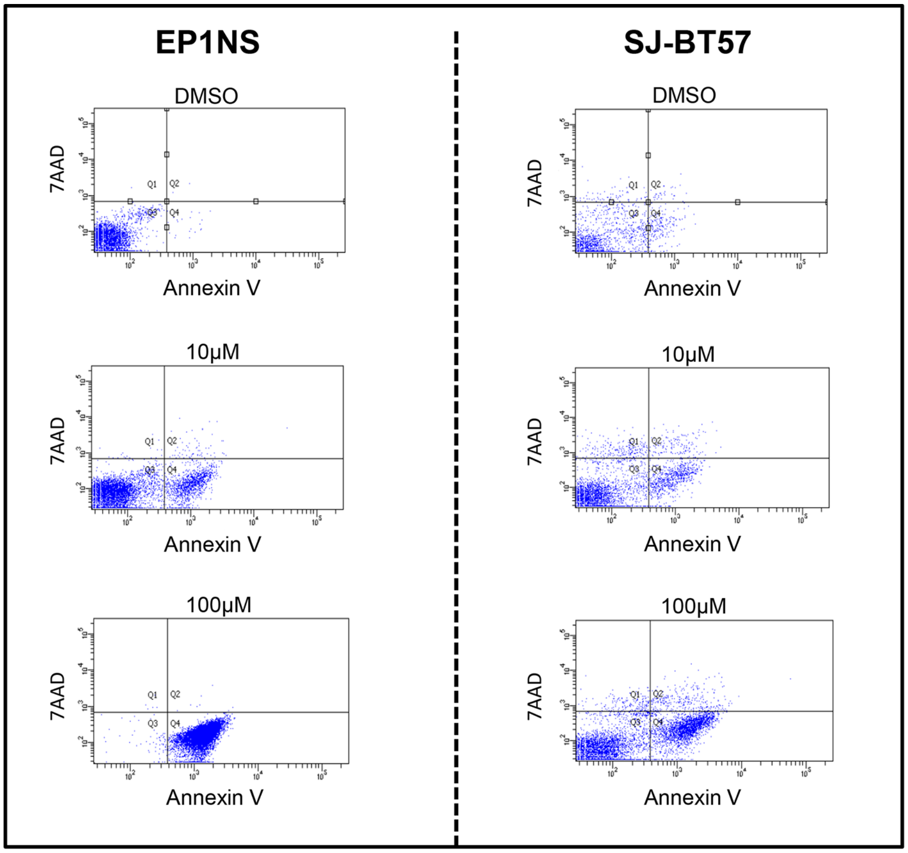

Supplementary Figure S8: Flow cytometric analysis after 7AAD and Annexin V staining of EP1NS (left panel) and SJ-BT57 (right panel) cells following 48 hours treatment with Nutlin-3. Illustrated are the DMSO controls, 10 µM and 100 µM Nutlin-3 treatments of the SJ-BT57 and EP1NS cells respectively.

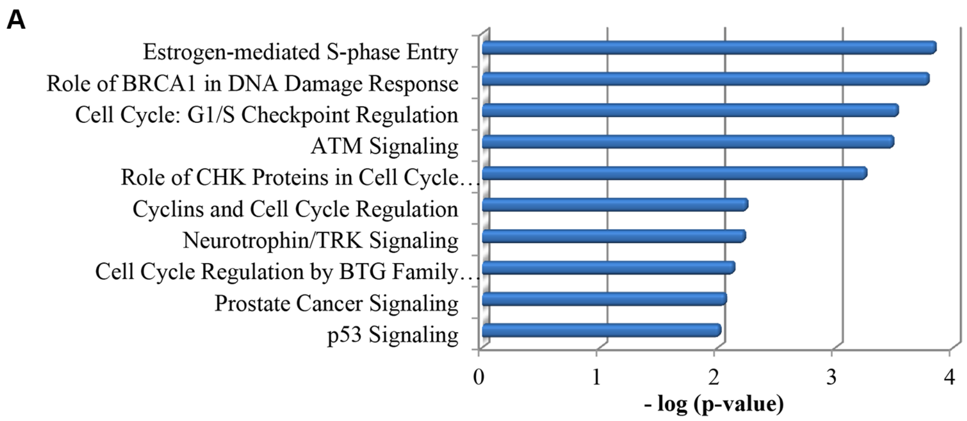

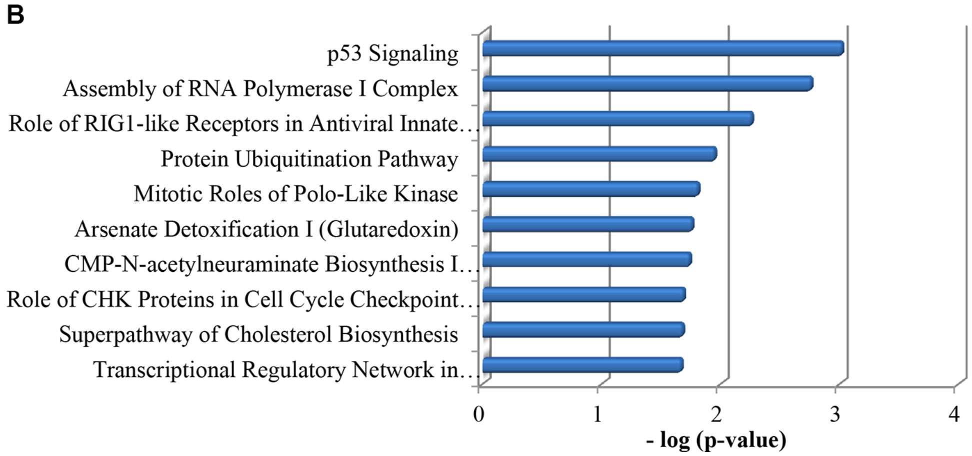

Supplementary Figure S9: Ingenuity pathway analysis of significant differentially regulated genes between high-dose (100 nM) and low-dose (5 nM) Actinomycin-D conducted separately for both cell lines, (A) SJ-BT57 and (B) EP1NS. The p53 pathway proved to be the top upregulated pathway after low-dose treatment with the cytostatic agent in the EP1NS cells.

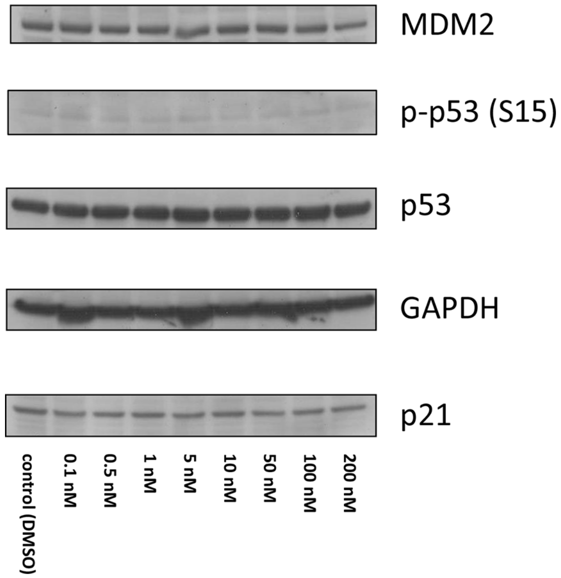

Supplementary Figure S10: Protein analysis of DAOY medulloblastoma cells after 6 hours treatment with Actinomycin-D confirming the absence of upregulation of p53-related genes and demonstrating constant levels of p53 in a *TP53* mutated cell line.

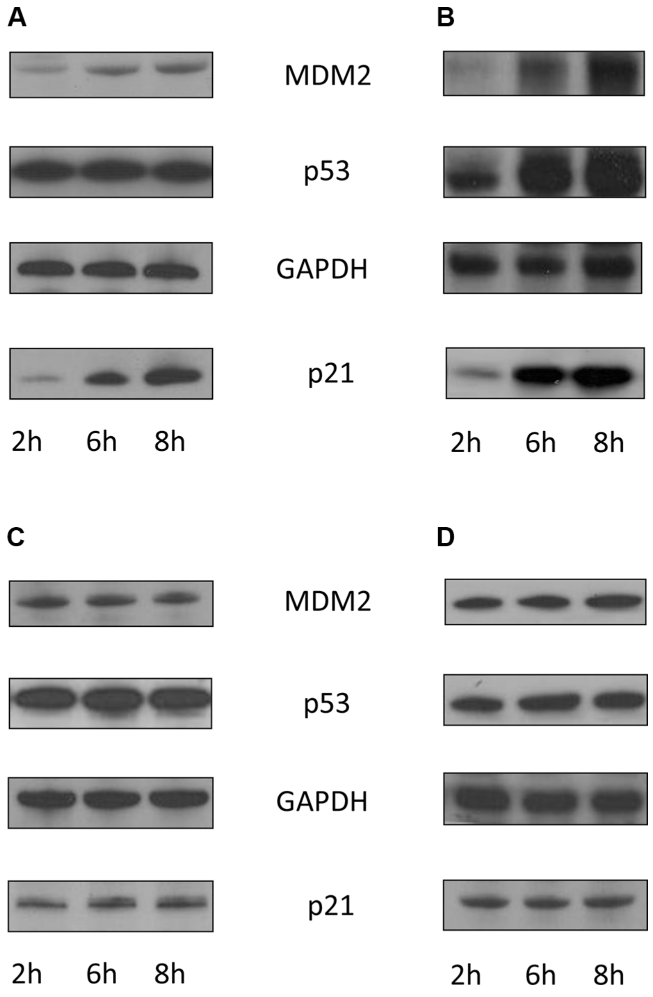

Supplementary Figure S11: Western blot analysis of 5 nM Actinomycin-D treated SJ-BT57 (A) and EP1NS (B) cells for 2 hours, 6 hours and 8 hours as well as their respective DMSO controls of SJ-BT57 (C) and EP1NS (D) cells indicating time-dependent upregulation of p53-associated partners after exposure of the cells to 5nM Actinomycin-D, while no difference is observed after DMSO treatment.

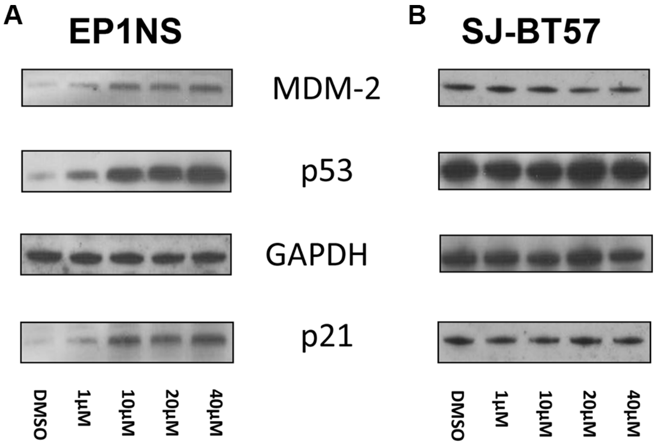

Supplementary Figure S12: Protein analysis of ependymoma cells after 6 hours treatment with Nutlin-3 showing a dose-dependent response upon the agent in the EP1NS, yet no effect in the SJ-BT57 cells.

Supplementary Table S1: p53 mutation in 130 primary tumors (4/130 = 3%)

Patient	mutation	exon	AA change	Exon number	Genomic description	CpG site	Splice site	WT base	WT codon	Mutant codon	Effect	location
EPN-005	g.del11045/46CT	2	–	2-exon	g.del11045/46CT	no	no	–	–	–	–	supratentorial
EPN-026	c.447C > T	5	p.S149S	5-exon	g.12435C > T	no	no	C	TCC	TCT	silent	infratentorial
EPN-031	c.509C > T	5	p.T170M	5-exon	g.12497C > T	yes	no	C	ACG	ATG	missense	infratentorial
EPN-030	c.743G > A	7	p.R248Q	7-exon	g.13380G > A	yes	no	G	CGG	CAG	missense	supratentorial

Supplementary Table S2: p53-associated differentially regulated genes in both cell lines (EP1NS, SJ-BT57) including the fold change value of low- versus high-dose Actinomycin-D treatment

**Both cell lines**		**SJ-BT57**		**EP1NS**	
**Molecular Subtype**		**PF-EPN-A**		**ST-EPN-RELA**	
**Gene**	**Fold change**	**Gene**	**Fold change**	**Gene**	**Fold change**
MDM2	6.239	*GADD45G*	5.864	***PUMA***	19.263
PUMA	5.916	*JMY*	5.838	*PIK3R1*	16.355
PIK3R1	5.524	*PML*	5.663	*GADD45A*	12.124
PIK3R3	4.557	*MDM2*	5.118	*FAS*	9.713
SIRT1	4.173	*TP53BP2*	4.864	*MDM2*	8.535
GADD45G	4.139	*SIRT1*	4.133	*PIK3C3*	8.454
PIK3C3	4.125	*PIK3R4*	3.948	*TP53INP1*	7.043
GADD45A	4.112	*MAPK14*	3.894	*CCNG1*	6.426
TP53BP2	4.025	*PIK3R1*	3.283	*PML*	6.044
JMY	3.897	*CHEK1*	3.262	*PIK3R3*	5.588
PIK3R4	3.785	*PIK3R3*	3.167	*TP53I3*	5.45
PTEN	3.306	*PTEN*	3.078	*C12orf5*	5.127
TP53I3	3.232	*PIK3C3*	2.926	*TNFRSF10B*	4.976
PML	3.091	*GNL3*	2.749	*DRAM1*	4.455
MAPK14	3.075	*PUMA*	2.596	*CDKN1A*	4.28
GNL3	3.062	*SNAI2*	2.157	*PTEN*	4.251
FAS	2.949	*PIK3CB*	2.049	*SIRT1*	4.245
C12orf5	2.903	*PIAS1*	2.015	*TNFRSF10A*	4.07
DRAM1	2.755			*CSNK1D*	3.982
SNAI2	2.711			*PCNA*	3.62
TNFRSF10B	2.54			*GNL3*	3.495
CHEK1	2.469			*PIK3R4*	3.49
CSNK1D	2.418			*SNAI2*	3.278
MED1	2.22			*RRM2B*	3.275
RRM2B	2.207			*SCO2*	3.226
				GADD45G	3.1
				ADCK3	3.073
				JMY	3.022
				MED1	2.997
				CCNK	2.857
				JUN	2.743
				HIF1A	2.558
				TP53BP2	2.517
				PMAIP1	2.507
				MAPK14	2.397
				RPRM	2.225
				PIK3CA	2.175
